# Supplementary material for: Psychosocial and behavioral correlates with HIV testing among men who have sex with men during the COVID‐19 pandemic in China
Source: PLoS One. 2022 Jan 24;17(1):e0262472. doi: 10.1371/journal.pone.0262472 (PMC8786173; doi:10.1371/journal.pone.0262472)
Supplement: Attachment — Submitted filename: Response to reviewers.docx [file pone.0262472.s001.docx]

Dear Editor,

Thank you for the opportunity to revise and resubmit our manuscript, “*Psychosocial and behavioral correlates with HIV testing among men who have sex with men during the COVID‐19 pandemic in China*”. The recommendations of the reviewer were constructive, and we have made appropriate changes. We enumerate the list of suggestions by the reviewer and provide our detailed responses below.

1. Please ensure that your manuscript meets PLOS ONE's style requirements, including those for file naming. The PLOS ONE style templates can be found at

https://journals.plos.org/plosone/s/file?id=wjVg/PLOSOne_formatting_sample_main_body.pdf and <https://journals.plos.org/plosone/s/file?id=ba62/PLOSOne_formatting_sample_title_authors_affiliations.pdf>

**Response: Thank you for the reminder.** Our manuscript and file names have met PLOS ONE’ style requirements.

2. Thank you for including your ethics statement: "The study was approved by the local human subjects research institutional review board (No. of IRB Application: JSJK2019-B012-02). All participants provided written informed consent."

a) Please amend your current ethics statement to include the full name of the ethics committee/institutional review board(s) that approved your specific study. Once you have amended this/these statement(s) in the Methods section of the manuscript, please add the same text to the “Ethics Statement” field of the submission form (via “Edit Submission”).

**Response:** The full name of the ethics committee/institutional review board has been included in the revised manuscript, i..e, “The study was approved by ethics committee of Jiangsu Provincial Center for Disease Prevention and Control (No. of IRB Application: JSJK2019-B012-02).” We also added the same text to the “Ethics statement” field of the submission form via “Edit submission”

3. We note that you have indicated that data from this study are available upon request. PLOS only allows data to be available upon request if there are legal or ethical restrictions on sharing data publicly. For more information on unacceptable data access restrictions, please see <http://journals.plos.org/plosone/s/data-availability#loc-unacceptable-data-access-restrictions>.

In your revised cover letter, please address the following prompts:

a) If there are ethical or legal restrictions on sharing a de-identified data set, please explain them in detail (e.g., data contain potentially sensitive information, data are owned by a third-party organization, etc.) and who has imposed them (e.g., an ethics committee). Please also provide contact information for a data access committee, ethics committee, or other institutional body to which data requests may be sent.

b) If there are no restrictions, please upload the minimal anonymized data set necessary to replicate your study findings as either Supporting Information files or to a stable, public repository and provide us with the relevant URLs, DOIs, or accession numbers. For a list of acceptable repositories, please see http://journals.plos.org/plosone/s/data-availability#loc-recommended-repositories.

We will update your Data Availability statement on your behalf to reflect the information you provide.

**Response:** We added the details about the data sharing as: “Data from this study are available upon request” in the revised cover letter. Jiangsu Provincial Center for Disease control and Prevention have the whole rights for this data. The data contain sensitive information. If you want to get the original data for study, please contact with the Corresponding author”.

4. Please include your tables as part of your main manuscript and remove the individual files. Please note that supplementary tables (should remain/ be uploaded) as separate "supporting information" files"

**Response:** The tables are now part of the main manuscript.

5. Reponses to Review Comments

**Reviewer #1:**

1) Methods: Is the survey nationwide or in the Jiangsu province. Line 139 says the province, but line 141 says nationwide.

**Response:** We now revised the statement: “Briefly, there were fourteen HIV sentinel sites located in thirteen cities in Jiangsu, and more than 6,000 MSM participated during the 2020 surveillance period.”

2) For eligibility criteria, did the men also have to be HIV negative?

**Response:** The participants do not have to be HIV negative.

3) The survey was face-to-face. That could impact response rates during a pandemic. Could you address this in the discussion? What was the response rate? Was there an incentive?

**Response:** This study was conducted after China lift the COVID-19 restriction. Considering we needed to collect the participants’ blood samples, participants had to complete the survey in person at the local health department. There was an incentive (50 RMB) for the participants who completed the whole study, including questionnaire and blood samples. Participants were recruited by the local health department. The response rate was 100%. We also addressed the limitation of the generatability of this sample in the revised Discussion.

4) Results: Remember to discuss results in past tense.

**Response:** We made changes in the revised Result.

5) Discussion: I think a big limitation in comparing testing rates between your survey and others is the different lengths of time that are assessed. This is addressed briefly, but should be discussed further. When you summarize the results for other studies you should include the time frame (i.e. percent tested within a 12-month period or 6-month period, etc). This is important information when comparing to your 3-month testing rate, since you would expect the proportion to be lower than the proportion testing in past 12-months. You should try to include results from other studies that also ask about a 3-month window for a more comparable comparison.

**Response:** We appreciate this insightful comment. We were able to access the annual national HIV sentinel surveillance data for the past few years and identify the HIV testing rates during the same 3-month window in previous years. Now we included the following discussion in the revised manuscript: “According to the annual national HIV sentinel surveillance data, the HIV testing rates among MSM were between 57% to 68% during the same 3-month window from 2016 to 2019 in Jiangsu (unpublished data). A significant decline in HIV testing among MSM during the COVID-19 state-enforced quarantine were observed in our study. The testing rate in our study was much lower than what were reported in studies prior to the COVID-19 pandemic, when the lifetime testing rates were 54% or the testing rate during the past 12 months were 56%-68%.”

6) Tables: In Table 2 you don't need to include both the did test and didn't test columns since you one is the inverse of the other. I would only include the "did test". You can also add a total column and a col percent column, so then most of the info in Table 1 can be shown in Table 2. Table 1 then can just show location of test among those that tested, and the the results of the three questions asked of non-testers. You should also make it clear that those three questions are only asked of the non-testers, so it is clear why it's a smaller sample size.

**Response:** We revised the tables based on the reviewer’s suggestion.

7) The description of the location in the title of the table should also be consistent between the two tables.

**Response:** The descriptions of the location in the both titles are the same now.

**Reviewer #2:** Apart from all other issues in this paper, I do not think it is possible to to analyze the impact of state enforced Covid-19 quarantine on HIV testing if one of the enrollment criteria was "have not taken any HIV test during the past 12 months" (line 145-146). An association between enrollment criteria and the outcome of the study is a serious fallacy. Moreover, the past 12 months also cover the period of covid state enforced quarantine. Hence the impact thereof cannot be evaluated in this study.

**Response:** Thanks for the comments. The inclusion criteria for this study did not include the part of “have not taken any HIV test during the past 12 months.” We apologize for the confusion.

Thank you again for the opportunity to revise and resubmit our manuscript. We have found the reviewer’s comments to be helpful and believe that we have answered all of the major concerns and comments. Please contact us if there are any other concerns. We look forward to working with you.
